# Determination of Rutin in Cigarette Tobacco, Filters, Mainstream Smoke and Burned Ash of Different Branded Cigarettes by High Performance Liquid Chromatography

**DOI:** 10.3390/molecules17043751

**Published:** 2012-03-26

**Authors:** Yinshi Sun, Wei Li, Jianhua Wang, Jianjie Bi, Shudong Su

**Affiliations:** 1 State Key Laboratory of Crop Biology, Shandong Key Laboratory of Crop Biology, College of Agronomy, Shandong Agricultural University, Taian 271018, China; 2 College of Chinese Medicinal Materials, Jilin Agricultural University, Changchun 130118, China

**Keywords:** quantitative analysis, rutin, tobacco, filter, mainstream smoke, burned ash, HPLC

## Abstract

Tobacco consists of at least 3,800 chemical constituents. Among them, rutin is an important polyphenolic secondary metabolite in tobacco, which has positive actions such as antiallergic, anti-inflammatory and vasoactive, antitumor, antibacterial, antiviral and anti-protozoal properties. A high performance liquid chromatography method was used to analyze rutin in tobacco and filters, mainstream smoke, and burned ash of ten varieties of cigarettes made in China. The chromatographic analysis was performed on a Hypersil ODS2 column with a gradient elution of acetonitrile and water at a flow rate of 1.0 mL/min. Detection was carried out at 350 nm using a photodiode array detector. The calibration curves for the determination of analytes showed good linearity over the investigated ranges (*R^2^* > 0.9998). Precision and reproducibility were evaluated by six replicated analyses, and the R.S.D. values were less than 0.59% and 1.53%. The recoveries were between 98.47 and 100.84%. Under the optimized conditions, namely 45 mL/g of solvent to solid ratio, 30 min of extraction time and 200 W of ultrasound power, the concentrations of rutin in tobacco and filter, mainstream smoke, burned ash of different brands cigarettes were 10.20–63.98, 0.10–0.32, 0.06–0.16 and 0 μg/per cigarette, respectively.

## 1. Introduction

It is well known that the cigarette smoking is a major source of particles in indoor air and potentially causes human respiratory diseases, due to the presence of hazardous substances in tobacco smoke, e.g., trace metals, carbon monoxide and nicotine [1,2,3,4]. However, the presence of some useful components including organic acids and polyphenols, is often overlooked [5,6]. Organic acids are the main flavor components of tobacco. On the other hand, polyphenols, widely distributed in tobacco plants and products, play an important role during the processes of physiological metabolism of tobacco. Polyphenols are considered vital components of tobacco in view of their contribution to sensory properties-flavor, color, bitterness, and antioxidant properties. Rutin, one of the major polyphenol components of tobacco, can indirectly affect the quality of tobacco [7,8,9]. Rutin exhibits many pharmacological activities including antiallergic [10], anti-inflammatory and vasoactive [11], antitumor [12], antibacterial, antiviral and anti-protozoal properties [13]. Although many researchers have paid extensive attention to rutin in tobacco, few reports have attempted to investigate the dynamic changes of rutin content in tobacco and during smoke delivery. Due to the considerable effects of rutin, this study investigates the rutin content in tobacco and filters, mainstream smoke and burned ash of different brands of cigarettes.

## 2. Results and Discussions

### 2.1. Optimization of the Solvent to Solid Ratio

**Figure 1:**
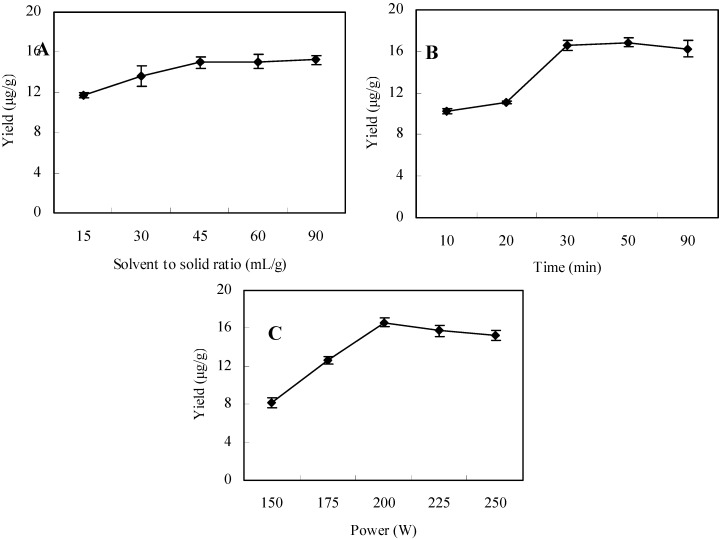
Effect of extraction conditions on the yield of rutin.

Generally, a larger solvent volume can dissolve constituents more effectively, leading to an improved extraction yield [14,15], but this will lead to a cumbersome concentration process and solvent waste. On the other hand, addition of a small amount of solvent will result in the lower yields of the objective constituents [16,17]. In this study, the solvent to solid ratio was investigated in the range of 15–90 mL/g. As shown in Figure 1A, by increasing the solvent to solid ratio, the extraction yields were increased. When the solvent to solid ratio increased over 45 mL/g, there are no significant differences, so 45 mL/g was selected as the optimized solvent to solid ratio.

### 2.2. Optimization of the Extraction Time

Data shown in Figure 1B indicated an obvious increase of extraction yield of the rutin when the time was increased from 10 to 30 min. When the time was increased from 30 to 90 min, however, no significant differences between the extraction yields of rutin (*p* > 0.05) was detected. Considering that shorter extraction times could lead to incomplete extraction and longer extraction times could result in time and solvent waste, 30 min was eventually selected as the optimal extraction time.

### 2.3. Effect of Ultrasound Power

The effect of ultrasound power on UAE was explored with a solvent to solid ratio of 45 mL/g and extraction time of 30 min. As shown in Figure 1C, the highest extraction yield of rutin was obtained at an ultrasound power of 200 W. When the ultrasound power was above 200 W, the extraction yield for rutin decreased. Therefore, ultrasound power of 200 W was deemed suitable for the extraction.

### 2.4. Optimization of HPLC Method

**Figure 2:**
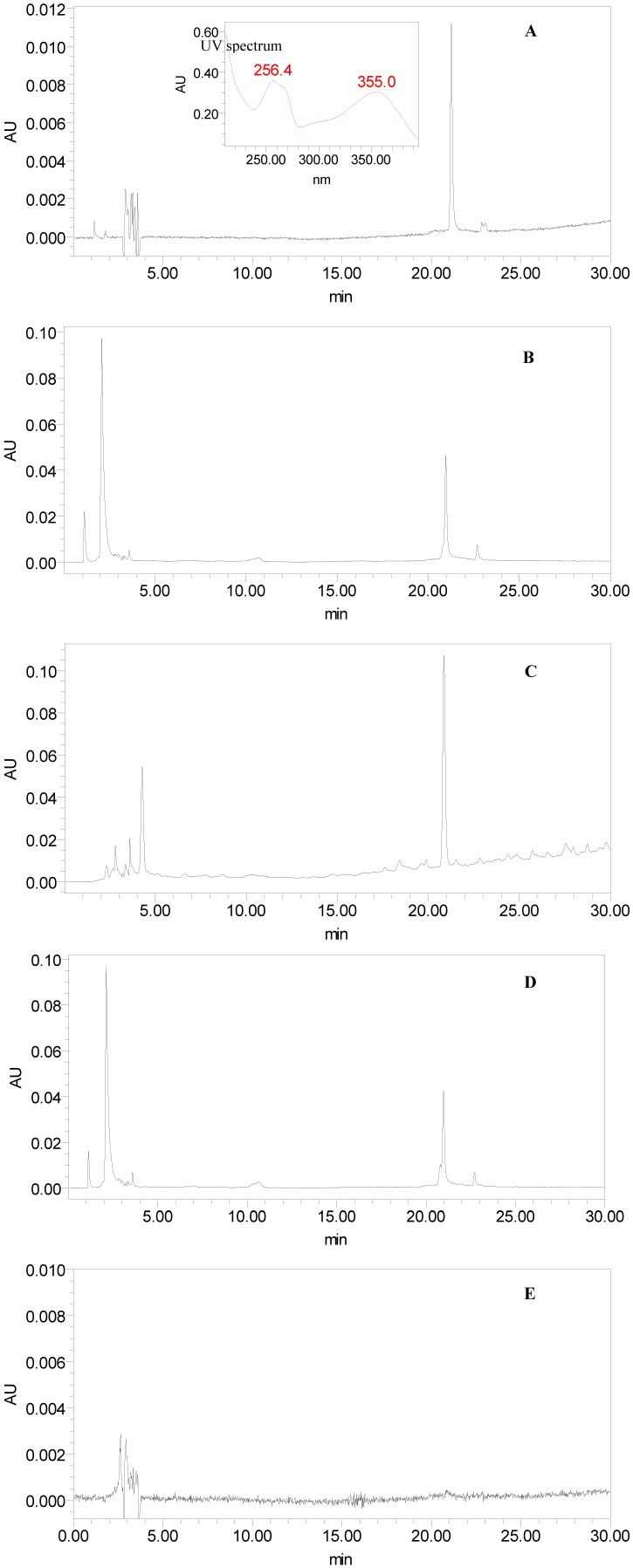
The HPLC chromatograms of the standard solution and brand 2 samples. (**A**) standard solution of rutin; (**B**) tobacco; (**C**) filter; (**D**) mainstream smoke; (**E**) burned ash.

In the present work, the HPLC method for the analysis of the crude sample was established first. In order to select an appropriate elution system for the HPLC separation of crude sample, different kinds of solvents (methanol–water, acetonitrile–water) were employed. The results indicated that when acetonitrile–water was used as the mobile phase, major peaks can be observed and each peak had baseline separation. Next the separation conditions of the analytes were optimized by systematically adjusting the acetonitrile content in the mobile phase. Figure 2A shows the standard substance with retention time of 21 min for rutin. Figure 2B–E are the HPLC chromatograms of tobacco, filter, smoke and burned ash of a typical brand of cigarettes.

### 2.5. Preparation of Calibration Curve

A series of standard solution with six different concentrations were analyzed by the established method in triplicate with an injection volume of 20 μL. The calibration curve for rutin was constructed by plotting the peak area (*y*) versus the concentration of standard analyte (*x*, mg/mL). The linear regression equations, correlation coefficients (*R^2^*) and linearity ranges The limit of detection (LOD) and limit of quantification (LOQ) were the concentrations of a compound at which its signal-to-noise ratios (*S/N*) were detected as 3:1 and 10:1, respectively. The LOD and LOQ values are listed in Table 1.

**Table 1:** Linear regression equation, correlationcoefficient and linearity of rutin.

Compound	Regression equation	*R^2^*	Linearity range (mg/mL)	LOD (μg/mL)	LOQ (μg/mL)
rutin	*y* = 17438 *x* – 24155	0.9998	0.03–0.93	10.88	32.63

### 2.6. Precision

The precision of the method was determined by the analysis of six consecutive injections using the same standard solution (0.06 mg/mL). The relative standard deviation (R.S.D.) for rutin peak areas was 0.59% (*n *= 6).

### 2.7. Reproducibility Test

Reproducibility of tobacco was evaluated by six replicated analyses. The R.S.D.s. for rutin level in six replicated tobacco measurements was 1.53% (*n *= 6). Data shown indicated the HPLC had good reproducibility for determination of the rutin in tobacco.

### 2.8. Recovery Test

In the recovery test, three different concentrations of rutin were added to known amounts of the pre-analyzed tobacco sample solutions, and then the spiked samples were analyzed three times (*n* = 3) by the established HPLC method. The results are listed in Table 2. The average recoveries for rutin were 98.47–100.84%, and their R.S.D.s. were less than 2.64%. Therefore, the HPLC method is precise, accurate and sensitive enough for quantitative evaluation of rutin in tobacco.

**Table 2:** Recovery test of the rutin fromtobacco (*n *= 3).

Component	Original amount (μg)	Amount spiked (μg)	Amount found (μg)	peak height 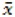 (μv)	Standard deviation σ (min)	Recovery (%)	R.S.D. (%)
rutin	4.15 ± 0.11	3.26 ± 0.13	7.39 ± 0.23	36985 ± 866	0.17 ± 0.01	99.19 ± 0.35	1.58 ± 0.21
	4.15 ± 0.11	4.18 ± 0.20	8.37 ± 0.19	42153 ± 539	0.25 ± 0.02	100.84 ± 0.47	2.64 ± 0.91
	4.15 ± 0.11	4.93 ± 0.16	9.01 ± 0.50	43379 ± 933	0.24 ± 0.02	98.47 ± 0.34	2.29 ± 0.59

The data has been presented as average of three determinations; Recovery (%) = 100 × (amount found – original amount)/amount spiked.

**Table 3:** Amount of rutin in tobacco, filter, smoke and burned ashof ten brands cigarettes (*n *= 3).

Brands	Contents (μg/per cigarette)			
	Tobacco	Filter	Mainstream smoke	Burned ash
brand 1	49.42 ± 0.90 b	0.19 ± 0.01 d	0.14 ± 0.00 b	ND
brand 2	20.76 ± 0.64 d	0.23 ± 0.02 c	0.13 ± 0.01 bc	ND
brand 3	20.84 ± 0.75 d	0.14 ±0.00 f	0.06 ± 0.01 e	ND
brand 4	15.55 ± 1.25 e	0.22 ± 0.01 c	0.12 ± 0.01 c	ND
brand 5	27.57 ± 1.03 c	0.26 ± 0.01 b	0.08 ± 0.01 d	ND
brand 6	17.38 ± 1.34 e	0.32 ± 0.01 a	0.16 ± 0.02 a	ND
brand 7	10.20 ± 0.30 f	0.15 ± 0.01 e	0.12 ± 0.01 c	ND
brand 8	29.21 ± 1.77 c	0.16 ± 0.01 e	0.08 ± 0.01 d	ND
brand 9	28.79 ± 1.16 c	0.14 ± 0.01 ef	0.08 ± 0.01 d	ND
brand 10	63.98 ± 2.43 a	0.10 ± 0.01 g	0.07 ± 0.01 de	ND

ND, not detected.

### 2.9. Application

The optimal conditions were applied to quantitative analysis of rutin in tobacco, filters, mainstream smoke and burned ash of ten varieties of cigarettes made in China and the results are presented in Table 3. The chromatogram of brand 2 sample is shown in Figure 2B–E. The identification of the investigated compounds was carried out by comparison of their retention time and UV spectra of the standard compound.

## 3. Experimental

### 3.1. Reagents and Materials

All organic solvents used for rutin extraction were of analytical grade and purchased from Tianjin Chemical Factory (Tianjin, China). Acetonitrile used for HPLC was of chromatographic grade (Fisher Scientific, Fair Lawn, NJ, USA). Water used was redistilled water, and passed through a 0.22 μm filter prior to use in all the studies. Rutin was purchased from the National Institute for the Control of Pharmaceutical and Biological Products (No. 2 Tiantan Xili, Chongwen District, Beijing) with a purity of over 98%. Packs of the ten varieties of Chinese made cigarettes were purchased from local convenience stores.

### 3.2. Instrument and Chromatography Conditions

The high performance liquid chromatography (HPLC) equipment used is a Waters 600E system (Waters, MA, USA) including a 4-solvent delivery system, 600E start-up kit and a 600 pump, 0–20 mL/min, a 2996 photodiode array detector, an Empower Add-on Single System (Waters, MA, USA), a 4-chamber in-line degasser and a 600E controller. The chromatographic analysis was performed on a Hypersil ODS column (250 mm × 4.6 mm i.d., 5 μm) at ambient room temperature with gradient elution of acetonitrile (A) and water (B) at a flow rate of 1.0 mL/min. A gradient program was used as follows: 22% A at 0–10 min and 22–61% A at 10–35 min. Detection was carried out using a 2996 photodiode array detector at 350 nm.

### 3.3. Sample Preparation

*Tobacco samples*: The cigarette tobacco and filter were separated and dried at 60 °C in a vacuum oven. Then, tobacco (0.2 g) was extracted with methanol (45 mL/g of solvent to solid ratio and in an ultrasonic water bath (200 W of ultrasound power) for 30 min. After the extraction procedure, the filtered solutions were concentrated to dryness in vacuum at 45 °C. The obtained dry extracts were diluted in methanol (50 mL).

**Figure 3:**
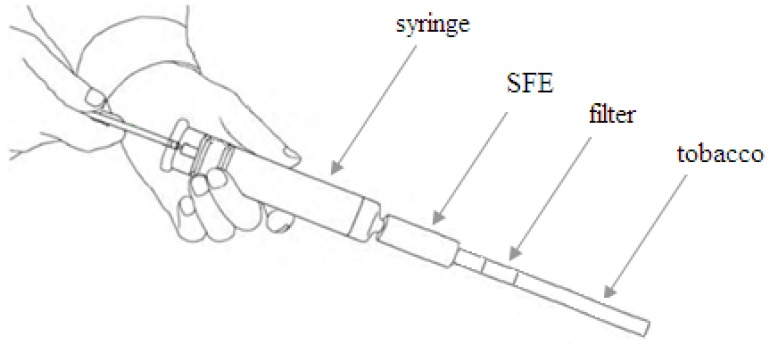
The installationfor simulating human smoking.

*Filter and burned ash samples*: A smoking installation (Figure 3) was used for simulating human smoking with settings of 1 puff per minute, puff volume of 35 mL, and puff duration of 2 seconds [18,19]. After the smoke procedure, the collected filter and burned ash were extracted and the filtered solutions were concentrated to dryness in vacuum at 45 °C. The obtained dry extracts were diluted in methanol (2 mL).

*Mainstream smoke samples*: After the smoke procedure, the C18 solid phase extraction (SPE) column was eluted with methanol (50 mL), the filtered solutions were concentrated to dryness, and then the dry extracts were diluted in methanol (2 mL).

All solutions were filtered through 0.45 μm membrane filter before direct injection into the HPLC system.

### 3.4. Preparation of Standard Solution

Stock solution was prepared by dissolving rutin (14.5 mg) in methanol (50 mL). Then, the solution was diluted step by step with methanol to give six different concentrations of working standard solutions to establish calibration curves. All solutions were filtered through 0.45 μm membrane filter prior to analysis.

## 4. Conclusions

In the present paper, a HPLC method was employed to detected rutin content in tobacco. The results indicated that the considerable amount of rutin ranging from 10.20 to 63.98 μg/per cigarette was found. However, only a low amount of rutin was found in filters and mainstream smoke with a content range of 0.10–0.32 μg and 0.06–0.16 μg/per cigarette, respectively. Rutin was not detected in burned ash, probably because that rutin was degraded after burning, which is supported by the results of previous studies [20]. Eventually, although only a small amount of rutin was inhaled into the human body when smoking a cigarette, to a certain degree, may have a positive effect for those who consume several cigarettes daily due to its accumulation effect. Due to the positive effects of rutin in tobacco, it is necessary to investigate how rutin was transferred and absorbed in our further work. 
